# The Tyranny of Distance and Impacts on an Ageing Australia

**DOI:** 10.1002/nop2.70138

**Published:** 2025-01-13

**Authors:** Scott William, Patricia M. Davidson

**Affiliations:** ^1^ Centre for Chronic and Complex Care Research, Blacktown Hospital, Western Sydney Local Health District Blacktown New South Wales Australia; ^2^ School of Nursing University of Wollongong Wollongong New South Wales Australia; ^3^ University of NSW Sydney New South Wales Australia

Globally we are experiencing rapid demographic shifts where populations are ageing. These demographic shifts are predicted to be more pronounced in rural, regional and remote communities. Australia has a vast landmass with a population of 26,699,482. The population is ageing, the fertility rate is falling, and in 2020, 16% of Australians were over the age of 65 years compared to 12% in 1990. These demographic trends are projected to continue. Approximately 30% of Australians are living outside of major cities and metropolitan areas and instead living in rural, regional or remote communities and these individuals experience inferior health outcomes to their urban counterparts (Australian Institute of Health and Welfare 2024). It is likely that this population growth will continue outside of major cities and metropolitan areas with a rising ageing population (Australian bureau of Statistics 2024).

Increases in the cost of living and a housing crisis have driven a population shift beyond the cities to regional communities. Simultaneously we are confronted by several workforce and industry shortcomings which is also partly due to an ageing workforce, a housing crisis and a potential for greater and sustained internal migration from non‐metropolitan areas. The proportion of older Australians participating in the workforce has doubled from 1971 to 2021 (Australian Bureau of Statistics 2022). Australia is prone to natural disasters. Natural disasters can commonly occur outside of major population centres and include wildfires, floods, severe storm events and cyclones. These natural disasters are known to impact transport networks, essential service accessibility and increase the need for social and healthcare access for Australians living in affected isolated or dispersed communities. Given that transportation, housing, employment and service accessibility are significant social determinants of health, these crises combined represent considerable headwinds.

Australia is facing multifactorial levels of economic uncertainty resulting from a cost‐of‐living crisis, workforce shortages and a national housing crisis. The notable workforce shortages extend beyond health and include trades personnel, transport, logistics workers and other essential services. The results of the cost of living and housing crises mean that larger proportions of Australians may choose to leave the capital cities and their homes to live in rural, regional and remote areas of Australia. In rural, regional and remote Australian healthcare the level of accessibility contributes to a range of health disparities (Clark et al. 2012). The Cardiac Accessibility and Remoteness Index for Australia (ARIA) index provides insights into the disparities in health care for rural, regional and remote Australia, particularly for cardiovascular events (Clark et al. 2012). A significant proportion of this inequality relates not only to medical expertise and infrastructure (Clark et al. 2012), it also relates to the underlying transport infrastructure resulting from a disproportionately distributed population relative to the large landmass of the country and limited investment. A recent systematic review identified that although almost 30% of Australians live in rural, regional or remote areas (Australian Institute of Health and Welfare 2024), limited research has been undertaken to address their healthcare access needs (Wood et al. 2023).

Australia utilises more nursing home care services than 11 other OCED countries for person over the age of 80 (Dyer et al. 2020). However, there are serious questions regarding the sustainability of the current model of aged care services as opposed to other flexible and innovative methods, such as in elder care services available at home and in the community. Although the health workforce in Australia is recorded as becoming younger, the healthcare workforce of rural, regional and remote Australia is ageing (Cortie et al. 2024). Furthermore, rural, regional and remote Australia faces significant shortages for aged care services which are projected to continue overtime (Blackberry and Morris 2023). These disparities are experienced by Aboriginal or Torres Strait Islander populations at a higher level than other Australians (Blackberry and Morris 2023). Enabling older Australians who live outside of major cities and metropolitans to live independently requires a strategic investment in developing key infrastructure.

Recently, the Australian Government has published its 23 recommendations in response to the outcomes of the Aged Care Taskforce, which highlight the need to ensure safe, equitable and cost‐effective aged care services for older Australians. Addressing the recommendations of the Aged Care Taskforce aligns with the Australian Infrastructure Plan 2021, and the United Nations Sustainable Development Goals. Understanding the interrelation of these recommendations to address key determinants of health is pertinent to enabling older Australians who would like to sustain a level of independence. These recommendations put forward a foundation which if pursued would enable a more sustainable future for older Australian's who choose to live outside of major cities. This will involve innovative, coordinated workforce strategies and investment in infrastructure, both capital and digital, through policy driven decisions backed by both the public and private sectors. Figure 1 provides a graphical representation of the solutions required to address the mentioned social determinants of health.

**FIGURE 1:**
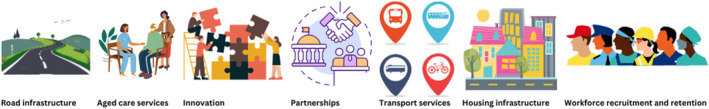
Graphical representation of solutions to reduce the tyranny of distance Australian's receiving equitable healthcare.

Therefore, addressing infrastructure, housing and essential services through a combination of public and private partnerships to improve healthcare access is critical. Working towards bridging these gaps is critical to improving health outcomes. Potential solutions to addressing these barriers include community engagement, development of key infrastructure such as roads, public transportation, community development to entice a younger workforce, internet and communications infrastructure, telehealth services, support for regional education and clinical education, and the further development of regional health centres. Advances in policy and implementation to practice through strategic partnerships should empower health professionals to deliver best‐practice care to Australians living outside of major cities. Nurses and other health professionals need to leverage technological innovation, such as telehealth as well as consider methods of increasing recruitment to non‐metropolitan areas through incentives and mentoring programs. Importantly, in policy, practice, education and research we need to address the powerful social determinants of health that drive health outcomes.

## 1 Conclusion

Increasingly there is a need to focus on the social determinants of health. Housing, access to essential services, supporting infrastructure development and transportation are critical elements in ensuring safe and equitable healthcare accessibility. As our population ages it is paramount for decision‐makers to focus on these issues with the wishes and preferences of older Australians in mind, to ensure greater synergy and congruency between infrastructure, health and essential services. Recognising the impact of living in rural, regional and regional areas is a critical first step.

## Conflicts of Interest

The authors declare no conflicts of interest.
